# American Medical Association

**Published:** 1854-05

**Authors:** 


### AMERICAN MEDICAL ASSOCIATION.

The American Medical Association met at St. Louis on the first Tuesday in this month, and after transacting the usual business, adjourned to meet, next year, in Philadelphia. We have not yet seen a full account of the proceedings, but learn from members who were present, that the most exciting question at the late meeting was the motion to transfer, from Philadelphia to New York, the printing of the Association's Transactions. The motion prevailed, but it was at the same time resolved that after the present year the transactions shall be printed at the place of meeting, and consequently they will be printed in Philadelphia in 1855. Dr. C. A. Pope, of St. Louis, was elected President of the Association. The entertainment provided for the Association by the Physicians of St. Louis is described as having been sumptuous in the extreme, and the private hospitality to the members equal to anything yet exhibited in any city where the Association has met. We hope in our next number to be able to give all the particulars of this interesting meeting.

### CHOLERA.

According to the last accounts from Paris cholera was on the increase in that city, the cases being more fatal as well as more numerous.

In New Albany a number of cases of cholera appeared towards the latter end of April, but for several weeks past we have heard nothing of the disease. A physician of that city informed us that it first broke out in the same houses in which it first made its appearance, in 1849, and just about the same season; and it is worthy of note that these houses are built in the immediate neighborhood of a filthy pond, one or two of them directly over it. The cases are said to have been twelve or thirteen, and we were told by our informant that all but one or two terminated fatally. Some of the friends of the families afflicted, who remained with the sick as nurses during the night, are reported to have suffered with the disease subsequently, but it did not spread or become epidemic.

